# Editorial: Wearable sensors role in promoting health and wellness via reliable and longitudinal monitoring

**DOI:** 10.3389/fphys.2023.1201847

**Published:** 2023-05-09

**Authors:** Sofia Scataglini, Thivya Kandappu, Avik Ghose, Aniruddha Sinha

**Affiliations:** ^1^ 4D4ALL Lab, Department of Rehabilitation Sciences and Physiotherapy, Center for Health and Technology (CHaT), Faculty of Medicine and Health Sciences, University of Antwerp, Antwerp, Belgium; ^2^ Computer Science Department in School of Computing and Information Systems, Singapore Management University, Singapore, Singapore; ^3^ TCS Research, Tata Consultancy Services (India), Kolkata, West Bengal, India

**Keywords:** health and wellness, wearables, multi-sensor fusion, digital biomarkers, bio-monitoring

Wearable technology has shown to be a crucial tool for reliable and long-term monitoring. The aim of this monitoring effort is to identify early biomarkers in physiological, physical, and biochemical profiles, such as heart rate (HR), HR Variability, breathing rate, oxygen saturation, that can be used for encouraging supposedly healthy people to change their lifestyle and improve their wellness and (ii) assisting clinicians by providing objective measures of patient’s state in daily activities.

The challenge of this human centered approach includes the design of non-invasive sensors for user-friendly and unobtrusive wearables, handling the noisy sensor data obtained in uncontrolled environment, performing multi-sensor fusion for reliable inferences and AI based analytics on longitudinal sensor data. According to the World Health Organization (WHO), health is a state of physical, mental and social wellbeing and not just the absence of disease. Cardiovascular disease (CVD), neurodegenerative diseases, diabetes, and cancer are among the leading causes of mortality and morbidity, but the causes and origins of these diseases are often controversial. Therefore, it is important to recognize early signs of stress, such as changes in physiological parameters that can be related to depression, to encourage people to lead healthier lifestyles.

Electroencephalogram (EEG) signals can serve to detect depression by using multi-channel data fusion and clipping augmentation and convolutional neural network as discussed by Wang et al., According to experimental findings, the combination of multichannel fusion (MCF) and multiscale clipping (MSC) enhancement can fully utilize the data present in individual sensor recordings and considerably enhance the classification accuracy and clustering effect of the diagnosis of depression.

Sleep is also an important factor that can affect both the physical health and mental wellbeing. Infact, stress and anxiety could also lead to disturbance in sleep. The study by Fan et al., demonstrates the feasibility of assessment of sleep stages and related autonomous nervous activity using the blood flow sensor which is based on laser doppler flowmetry and a mobile phone application. The duration and cycle of different sleep stages (REM, N1, N2, N3) characterises the quality of sleep. Monitoring of abnormal changes in blood pressure in daily living scenario, could prevent pre-hypertensive individuals to move to hypertensive condition through timely intervention. Blood pressure (BP) is regulated through a complex mechanism involving cardiovascular functions, renal functions, baroreflex control, and neural control of systemic circulation. Hu et al., presents a machine learning based approach for estimating BP using Photoplethysmogram (PPG) signals captured using wrist wearables. The improvement in the accuracy is obtained using Light Gradient Boosting approach which prevents the overfitting compared to traditional approaches like decision trees and random forests. Cuffless BP estimation algorithm is having immense potential, provided it can handle the demographic variations in PPG signals, which could only be known through large scale distribute trials. Physical, physiological and biochemical longitudinal data under everyday life conditions are also best collected in a non-intrusive manner using wearable sensors in neurorehabilitation.

Measurement of gait parameters, muscle forces, joint loading and range or motion are usually done in clinical settings using multi-camera setup and pressure sensors. Objective measures of the motor functions in neuromuscular disorders not only enable to quantify the progress of the disease, also helps in assessment of the therapeutic efficacy. Gerhalter et al., presents the use of smart watches to create a digital tool for measuring the mobility of upper limb and associated energy expenditure. Patients with neuromuscular diseases and healthy subjects participated in the study where the correlation is obtained between the movement and energy expenditure. This device is useful to quantify the slow movements and rotations of hand and arm which are crucial to perform daily living activities. Functional use of upper extremities is an important clinical measure for stroke patients. In matter of fact, the study by Geed et al., uses wrist wearable accelerometer sensors in patients with impaired hemiparetic arm, due to ischemic or haemorrhagic stroke. Random forest classifier is used to detect different types of functions like reach to grasp, hand gestures, door opening, which are performed with upper limbs. Accuracy of the classifier is validated against standard approaches like Action Research Arm Test, Nine-hole-peg test, Motor Activity Log. In medical investigations, postures inferred from recorded accelerations are frequently employed as indicators characterizing the activities of patients. However, when using consumer-grade wearable devices, recording for longer than 24 h is more likely to result in data losses than recording for a short period of time. Ogasawara et al., suggested an imputation technique that makes use of ensemble averaging to impute postures over a 24-hour period. By calculating the ratios of postures taken at the same time of day across many measurement-session days, this method generates a time series of postures spanning 24-hour with reduction in loss of data (proposing a solution considerably decreased the rate of missing data from 5.76% to 0.21%). The method was validated using 306 measurement data from 99 stroke inpatients in a hospital rehabilitation ward using an accelerometer positioned on the patient’s trunk.

This Research Topic aim to provide an explanation of the wearable sensor role for promoting health and wellness via reliable and longitudinal monitoring. The issue includes 6 papers addressed to use wearables for assessing health and wellness in different fields as in neurorehabilitation (e.g., Stroke and Parkinson Diseases), stress related diseases (e.g., using EEG diagnosis of depression or sleep disorder), for monitoring daily life activity as in ambient assisted leaving (AAL), or for monitoring physical, physiological and biochemical subject profile (e.g., posture, blood pressure, posture, muscle energy expenditure). All the contributions present an innovative way of using wearables as non-invasive way for monitoring physical, physiological, biochemical parameters providing clinicians a support for decision making and timely therapeutics.

